# Does diacritics‐based lexical disambiguation modulate word frequency, length, and predictability effects? An eye‐movements investigation of processing Arabic diacritics

**DOI:** 10.1371/journal.pone.0259987

**Published:** 2021-11-15

**Authors:** Ehab W. Hermena, Sana Bouamama, Simon P. Liversedge, Denis Drieghe

**Affiliations:** 1 Cognition and Neuroscience Research Laboratory, Department of Psychology, College of Natural and Health Sciences, Zayed University, Dubai, United Arab Emirates; 2 Centre for Perception and Cognition, Psychology, University of Southampton, Southampton, United Kingdom; 3 Perception, Cognition, and Neuroscience Group, School of Psychology, University of Central Lancashire, Preston, United Kingdom; Aix-Marseille Université, FRANCE

## Abstract

In Arabic, a predominantly consonantal script that features a high incidence of lexical ambiguity (heterophonic homographs), glyph-like marks called diacritics supply vowel information that clarifies how each consonant should be pronounced, and thereby disambiguate the pronunciation of consonantal strings. Diacritics are typically omitted from print except in situations where a particular homograph is not sufficiently disambiguated by the surrounding context. In three experiments we investigated whether the presence of disambiguating diacritics on target homographs modulates word frequency, length, and predictability effects during reading. In all experiments, the subordinate representation of the target homographs was instantiated by the diacritics (in the diacritized conditions), and by the context subsequent to the target homographs. The results replicated the effects of word frequency (Experiment 1), word length (Experiment 2), and predictability (Experiment 3). However, there was no evidence that diacritics-based disambiguation modulated these effects in the current study. Rather, diacritized targets in all experiments attracted longer first pass and later (go past and/or total fixation count) processing. These costs are suggested to be a manifestation of the subordinate bias effect. Furthermore, in all experiments, the diacritics-based disambiguation facilitated later sentence processing, relative to when the diacritics were absent. The reported findings expand existing knowledge about processing of diacritics, their contribution towards lexical ambiguity resolution, and sentence processing.

## Introduction

Arabic is a particularly interesting language for investigating how resolution of lexical ambiguity occurs, and how it influences reading behavior. This is because Arabic features a predominantly consonantal script, where each consonantal string can have multiple pronunciations, and meanings associated with these pronunciations (heterophonic homographs). As will be explained in more detail below, resolving lexical ambiguity associated with such words can be achieved by adding diacritics that convey vowel sound information, thus fully specifying the phonological and semantic representations of these words (e.g., the undiacritized letter string *قدر* /qdr/ which can be diacritized and pronounced as *قَدَرَ* /q^a^d^a^r^a^/; *قَدَرٌ* /q^a^d^a^r^un^/; *قَدَّرَ* /q^a^d^da^r^a^/; *قِدْرٌ* /q^i^dr^un^/, etc., with each pronunciation associated with a different meaning, see details below). In the absence of diacritics in everyday print, Arabic readers regularly rely upon context to disambiguate such words. We report three experiments that investigated diacritics-based lexical ambiguity resolution in different types of Arabic words, namely, words of high- and low-frequency (Experiment 1), short and long words (Experiment 2), and low-predictability words (Experiment 3, given that high-predictability words would not require such disambiguation).

### Word frequency, length, and predictability: The big three

Word frequency, length and predictability effects on eye movements, whereby high-frequency, short, or predictable words are read faster compared to low-frequency, longer, or unpredictable words, are considered benchmark findings in the reading literature, hence they are sometimes referred to as the ‘ big three’ [1, 2] (see [3, 4] for reviews). Numerous investigations have reported and replicated word frequency effects such that words that occur more frequently in a language attract shorter and fewer fixations and result in more skipping, compared to words that occur less frequently in the language (see, e.g., [5–7]). Likewise, Hermena and colleagues [8] reported that in Arabic, compared to low-frequency words, high-frequency words received a significantly shorter first pass reading time and also attracted significantly fewer first-pass fixations and a shorter go-past time (i.e., the sum of all fixation durations made from entering the target word region until exiting this region forward, including (re)fixations on preceding regions). Word frequency effects are typically explained as a function of repeated exposure to a word that results in increasing the speed with which the representation of this word is accessed and activated. Similarly, numerous investigations documented that words that contain more letters are skipped less often, attract longer fixation durations, and more fixations and re-fixations (see e.g., [9–15]). These findings of word length effects were also recently replicated in Arabic [16, 17]. Additionally, the findings reported in Arabic [16] further supported the idea that the number of letters a word contains modulates fixation durations, or the decision of *when* to move the eyes; whereas word skipping and other measures of *where* to move the eyes are influenced mainly by the word’s spatial extent, or the amount of horizontal space the word occupies (see also [18, 19]). The spatial extents of Arabic words vary, even for words that contain the same number of letters, because proportional fonts are typically used in Arabic print whereby letter sizes are allowed to vary.

Whereas word frequency and length are word-level variables, word predictability is a variable that reflects the degree to which a particular word is expected from the context that precedes it. A great deal of evidence shows that the predictability of a word affects eye movement behavior on that word with contextually predictable words (e.g., *cake* in: *The baker rushed the wedding cake to the ceremony*) yielding shorter fixation durations and more skipping compared to less predictable words that are equally semantically plausible (e.g., *pies* in: The *baker rushed the wedding pies to the ceremony*; see [10, 13, 20–27]). As yet, no published studies have documented word predictability effects in Arabic.

### Lexical ambiguity resolution: The case of Arabic

The omission of the vowel sounds from print in Arabic, as is the case also in Hebrew, is a feature of these Semitic languages [28]. Vowels are added in the form of diacritics to each letter, thus indicating how each consonantal letter string should be pronounced. For example, the letter string *قدر* /qdr/ can be pronounced as *قَدَرَ* /q^a^d^a^r^a^/ (*[he] was able to*, verb, past tense, masculine); *قَدَرٌ* /q^a^d^a^r^un^/ (*fate* or *destiny*, noun, masculine); *قَدَّرَ* /q^a^d^da^r^a^/ (*[he] estimated* or *destined*, verb, past tense, masculine); *قِدْرٌ* /q^i^dr^un^/ (*pot* or *vessel*, noun, masculine), etc. depending on the diacritization pattern the word is given. As these diacritics are typically removed from print, with the exception of educational materials for children up to 8–9 years of age, and some religious and literature texts [29, 30], the incidence of lexical ambiguity is high in Arabic, with one in every three words in normal text being an ambiguous heterophonic homograph, as in the example above [31]. Readers of Arabic become very apt in relying on context to disambiguate such homographs and to perform complete and accurate sentence comprehension [29–31]. It is also an established practice in Arabic print that diacritics may be added to a word in a sentence where the surrounding context does not sufficiently disambiguate the homograph, and thus diacritics can be added to such words in order to ‘locally’ remove the ambiguity on the otherwise ambiguous word itself. Arabic thus provides an ideal environment to investigate local (word-based) and context-based lexical disambiguation during text reading.

The lexically ambiguous heterophonic homographs in Arabic, as in the example above, are mostly *biased*, that is, have one dominant representation (phonological and associated semantic value). In the above example *قَدَرٌ* /q^a^d^a^r^un^/ *fate* (noun, masculine) is the dominant representation as it is more frequently encountered than other representations, whereas *قِدْرٌ* /q^i^dr^un^/ (*pot* or *vessel*, noun, masculine) can be thought of as the subordinate representation. Thus, the dominant and subordinate representations of the base orthographic form *قدر* /qdr/ are lexically different entries in terms of their phonological and semantic representations. Importantly, the presence of diacritics alters the orthographic representation of the word, thus instantiating a different word. In the absence of comprehensive databases that provides frequency counts for all diacritized versions of Arabic homographs, we are making the assumption that subordinate representations, instantiated by the diacritics, would actually be words of lower frequency than the dominant representations that readers would adopt when they encounter the undiacritized homographs. We are basing this assumption on the lower frequency with which these subordinate representations were produced during the norming procedure (see details below, see also [32] for further discussion). The fact that the diacritics-based disambiguation process instantiates a different word can be contrasted with homography in English and other languages, where such ambiguous words diverge only in their semantic representations, while sharing identical orthographic and phonological representations (e.g., *port*: a waterfront facility, as the dominant meaning, or a type of wine, as the subordinate meaning). The frequency difference between the dominant and subordinate representations is in the frequency one meaning or the other is instantiated by the same lexical entry, *port* [33].

Previous findings suggest that following a non-constraining context (i.e., a context that does not favor one particular meaning of the homograph over another), such biased homographs attract shorter fixation durations, relative to homographs that have two equally likely meanings, known as *balanced* homographs [34–37]. This is typically attributed to the costly competition between the equally likely word representations of the balanced homographs, whereas with biased homographs, the dominant analysis is accessed first with little competition from the subordinate representation(s).

Recent evidence showed that when reading a sentence that contains a biased homograph preceded by non-constraining context, Arabic readers adopt the dominant representation of that homograph, and later context would then serve to either confirm or to challenge the readers’ analysis. If subsequent context instantiates the subordinate representation of the homograph, and not the dominant representation adopted by the readers, disruption to processing is to be expected. Indeed, a recent investigation [38] found that in the absence of disambiguating context and diacritics, the readers adopted the dominant active voice representation of homographic Arabic verbs and significant disruption to processing occurred when subsequent context instantiated the subordinate, passive voice, representation of these verbs. Specifically, fixation durations (first pass and later re-reading measures) were inflated at the disambiguating region (after the target word) that instantiated the subordinate (passive voice) representation, and at the end of the sentence region, where readers typically perform final integration and synthesis processes (see e.g., [39]). These findings replicated what was reported in other languages, where readers experienced similar disruption to processing as they attempted to correct the inaccurate homograph representation they adopted, and sentence representation they constructed [34, 36, 37, 40–43].

The effect that the presence of diacritics has on reading performance has been studied in previous research. Some very informative investigations showed that readers depend heavily on the sentence and text context when reading undiacritized Arabic in reading aloud [29–31, 44]. Unsurprisingly, these studies showed that readers’ accuracy improved when diacritics were present. However, due to using off-line methodology (e.g., reporting accuracy rates), the nature of moment-to-moment processing of diacritics and diacritized words could not be inferred from these studies. Using on-line methods such as eye tracking, studies were equivocal with regards to the effect of homograph diacritization in sentence reading. In one study, there was little (and non-significant) difference between fixation durations on ambiguous verbs as a function of the presence or absence of the diacritics that disambiguated these verbs as passive [38]. On the other hand, using the boundary paradigm [45], where researchers manipulate what information is available to the readers about the upcoming word, that is, parafoveally, interesting findings were obtained regarding the effects of the diacritics being present on upcoming words. Typically, in boundary paradigm investigations, the presence of the target itself in the parafovea, known as ‘identity preview,’ results in processing facilitation (reduced fixation durations on the target) compared to when inaccurate or incomplete information about the target is presented parafoveally [3, 4]. In the case of Arabic, the presence of diacritics on an ambiguous target word located in the parafovea (i.e., typically the word following the fixated word), appeared to act as an early warning that the pronunciation of the upcoming diacritized word is likely to conform to the subordinate version [32]. Identity previews of the diacritics on the target word resulted in the typical preview benefit (reduced gaze duration) only for diacritics that instantiated the subordinate representation of the homograph, and not when the diacritics instantiated the dominant analysis. As such, whether the presence of disambiguating diacritics results in processing benefit may be contingent on whether the diacritization pattern instantiated the dominant or the subordinate representation of the target word: If the diacritics instantiate the latter, processing benefit (reduced first pass fixation durations) may be expected. Developing certain expectations about the information to be supplied by the diacritics is perhaps further evidence that readers’ experience with the language needs to be accommodated in lexical ambiguity resolution models (see e.g., [46]). Specifically, readers extract the statistical regularities about the co-occurrence of diacritics and the instantiation of the subordinate representation of homographs (almost all the time), and this appears to influence their eye movements during reading.

### The current experiments

In the current set of experiments, we aimed to expand what we know about the processing of Arabic diacritics. Specifically, we investigate whether adding diacritics to resolve lexical ambiguity, locally on the ambiguous word itself, would have similar or different effects on high- and low-frequency words, and on longer and shorter words, that is, if this mode of disambiguation would modulate these effects (Experiments 1 and 2). Additionally, we investigate whether the presence of disambiguating diacritics would facilitate the processing of words of low contextual predictability (Experiment 3).

In the reported experiments, biased ambiguous homographic Arabic words were embedded in sentences such that the context preceding these words did not disambiguate them, and subsequent context always instantiated the subordinate representation of these words. The words were presented either undiacritized, or carrying the diacritics that also always instantiated the subordinate representation.

### Experiments 1 and 2: Word frequency and word length and diacritics-based disambiguation

The experiments reported here aim to answer two questions. The first one is: How does diacritics-based disambiguation affect the processing of high- and low-frequency words, and short and long words? There are potentially multiple plausible scenarios to consider. To begin with, and on a simplistic level, it is possible that the presence of disambiguating diacritics will eliminate any competition between the different representations of the target homographs and thus facilitate processing of these target words. Although attractive, this scenario is not a likely one. Recall that evidence suggests that when processing biased homographs, such as the ones used as targets, readers access the dominant representation of this homograph with almost no competition from the other subordinate representations [34, 36]. If the diacritics-based disambiguation does indeed result in facilitation of processing the diacritized word, a more likely mechanism for this facilitation might proceed along the following lines. Readers would ‘spot’ the diacritics parafoveally, before fixating the target word, and this would cue the lexical processing system that, most likely, the subordinate meaning is being instantiated in the upcoming word, and thus the dominant representation is to be dismissed or suppressed. This may result in a head start in activating the subordinate representation of the homograph. Once the readers fixate the diacritized target, the subordinate analysis would be confirmed, in what we will refer to as the ‘spot-activate-verify’ mechanism. This may result in faster processing of the diacritized target words, and smoother progress in sentence reading. Importantly, if such benefit is obtained, it would indicate that the presence of the diacritics has successfully guided the readers towards a different lexical entry (the subordinate representation) from the entry the readers would access in the absence of diacritics (the dominant representation).

However, as mentioned above, it is rather unlikely that even if this mechanism of spotting the diacritics before fixating the word leads to facilitation, this facilitation would make processing the diacritized words (instantiating the subordinate representation) faster than processing the undiacritized words (the dominant representation is rapidly activated and assumed by the readers). Yet, it is hard to rule out this scenario completely given the available evidence that the presence of diacritics in the parafovea that instantiate the subordinate representation of homographs results in facilitation on the diacritized word itself [32], as well as the reported improved performance associated with the presence of diacritics in the text in other off-line investigations (see above).

An arguably more plausible scenario is informed by the classic findings of lexical ambiguity resolution research. Numerous studies reported significant processing costs when prior context disambiguates a biased homograph instantiating the subordinate analysis of this homograph. This has been referred to as the subordinate bias effect [34, 35, 40, 42, 47–49]. This effect is typically explained as the processing costs of having to suppress the dominant analysis of the homograph that is more readily accessible, in favor of the less-frequent, subordinate analysis [34, 35, 50]. Would the presence of diacritics that instantiate the subordinate analysis result in processing costs akin to the subordinate bias effect, given that readers would have to suppress the easily accessible dominant analysis of the homograph in favor of the subordinate analysis? If so, this would be an interesting instance of the subordinate bias effect and would suggest that this effect can be observed when the subordinate analysis of a homograph is instantiated on the homograph itself—the diacritized Arabic homograph, and not only when this subordinate analysis is instantiated by prior context. Note that Rayner et al. [42] were able to obtain a reliable subordinate bias effect when the word immediately before the ambiguous target (a modifier) instantiated the subordinate analysis of this target (e.g., the modifier *statistical* table vs. *kitchen* table). The use of diacritics in Arabic allows us to disambiguate the target word without any indications towards the subordinate meaning in the preceding context.

The second question these experiments aimed to investigate is would any facilitation, or costs, resulting from the presence of the diacritics affect high- and low-frequency words differently, such that an interaction between these variables would be observed? And the same question applies to the variables of word length (short, long) and diacritization (diacritized, undiacritized). As far as we know, if diacritics provide an early, parafoveal, phono-semantic cue to activate a particular pronunciation and meaning of the upcoming diacritized word, there are currently no theoretical frameworks that would predict that this particular process should affect high- or low-frequency words, or long and short words differently. The nature of this question, and the analyses of possible interactions between the variables of word frequency and length, and the presence of diacritics, are thus largely exploratory. With the diacritics available parafoveally, there are potentially two possibilities, with the diacritics acting as a pre-target cue to activate the subordinate representations and suppress the dominant ones: (a) Most likely, the diacritics on the upcoming word activate the subordinate *phonological* representation, and this leads to activation of the subordinate semantic representation (as in, e.g., the phonology-to-semantics route in the Dual Route Model [51]). Alternatively, (b) The diacritics activate the subordinate *semantic* representation of the upcoming homograph, and this would in turn activate its subordinate phonological representation (i.e., a semantics-to-phonology feedback route as in, e.g., the Triangle Model [52, also 51]). In either case, none of these models make explicit predictions regarding phono-semantic disambiguation that would differentially affect one type of words or another, particularly if the phono-semantic representations being instantiated are considerably less common (subordinate) than the word forms. It is more likely that if the presence of the disambiguating diacritics results in any facilitation or costs, these effects would be observable to a similar degree on high- and low-frequency words (Exp. 1), and on short and long words (Exp. 2). In the absence of definitive empirical evidence, however, exploring and documenting whether or not diacritics-based disambiguation modulates the effects of word frequency and length is one of the aims of this investigation.

Finally, and with regards to the effect of the presence of diacritics on sentence processing, in line with previous findings [38] readers’ eye movements at the end of sentence region, and particularly the re-reading of previous sections which originates from that region (go past measure) will also be examined and be used as an index of later integrative processes (see also [39]). If readers benefit from the presence of the disambiguating diacritics on the target, it is plausible to expect that as the rest of the sentence confirms the subordinate analysis of the target, there should be no disruption to processing. By contrast, if in the absence of diacritics readers do adopt the dominant representation of the homographic target, the subsequent sentence context will challenge this analysis and later integrative processes should reflect a degree of disruption.

### Method

#### Participants

The same set of participants took part in the eye tracking procedure in Experiments 1 and 2. The participants were forty-four native Arabic speakers (22 women; mean age = 31.0 years, SD = 6.2, range = 19–50) who participated in the eye tracking procedure after giving written informed consent.

In all three experiments, all participants had normal or corrected-to-normal vision. They were all recruited from the University of Southampton student population, and through the Arabic and Lebanese Society in Southampton, UK. The participants were compensated £15 each for participation.

*Participants for stimuli norming*. A total of thirty-six additional native Arabic speaking participants that did not take part in the eye tracking procedure were recruited (on-line) to perform the on-line norming tasks to prepare the stimuli used in all three experiments. These participants were from a number of Arab countries (incl. Algeria, Tunisia, and Jordan) and they were compensated £5 for their participation.

#### Stimuli

*Experiment 1*: *Word frequency × diacritization stimuli*. Twenty-eight pairs of high- and low-frequency words were selected from the Aralex corpus [53] as target words. High-frequency words had an average of 175 counts per million (CPM) in Aralex (SD = 7.5, range = 58.2–558.9), whereas the low-frequency words had an average of 3 CPM (SD = 129, range = 0.03–17.8). The difference of average log-transformed word frequency between the two groups was statistically significant *t*(54) = 7.0, *p* < .05. The high- and low-frequency word sets were matched on word length (for both sets, mean = 4.8 characters, SD = 2.9, range = 3–7). We used a proportional font (Traditional Arabic) where the natural size of Arabic letters vary in spatial extent (the horizontal space they occupy), which can result in words containing the same number of letters occupying different spatial extents [16]. To control for this potential confound, we used the same procedure described in previous studies [8, 16, 32] whereby we matched the high- and low-frequency word pairs of the same number of letters on spatial extent. Matching word pairs on spatial extent was achieved through extending letter ligatures when necessary by one or two pixels so that both words in a stimulus set would have the spatial extent of the largest one (see full details of this method in [16]). The target word pairs were also matched on average age of acquisition (see stimuli norming procedure below, mean _high-frequency_ = 9.1 years, SD = 1.0, range = 7–10.6; mean _low-frequency_ = 9.0 years, SD = 1, range = 7.0–10.8; *t*(54) < 1). The high- and low-frequency words were used either undiacritized or with the diacritics that instantiated the subordinate pronunciation. It is important to note that the undiacritized and diacritized words (in both frequency conditions) would instantiate the same pronunciation once placed in a sentence.

To make the use of diacritics on the target words ecologically valid, all target words, in all three experiments were: (a) heterophonic-homographs, that is ambiguous words the exact pronunciation of which requires sentence context or diacritics to access a full and accurate phono-semantic representation [38], (b) the sentence context preceding these homographs did not disambiguate them, and (c) as will be detailed below, the correct pronunciation of the selected target words corresponded to one of the subordinate pronunciations possible for the letter string [32, p.2023).

**Fig 1:**
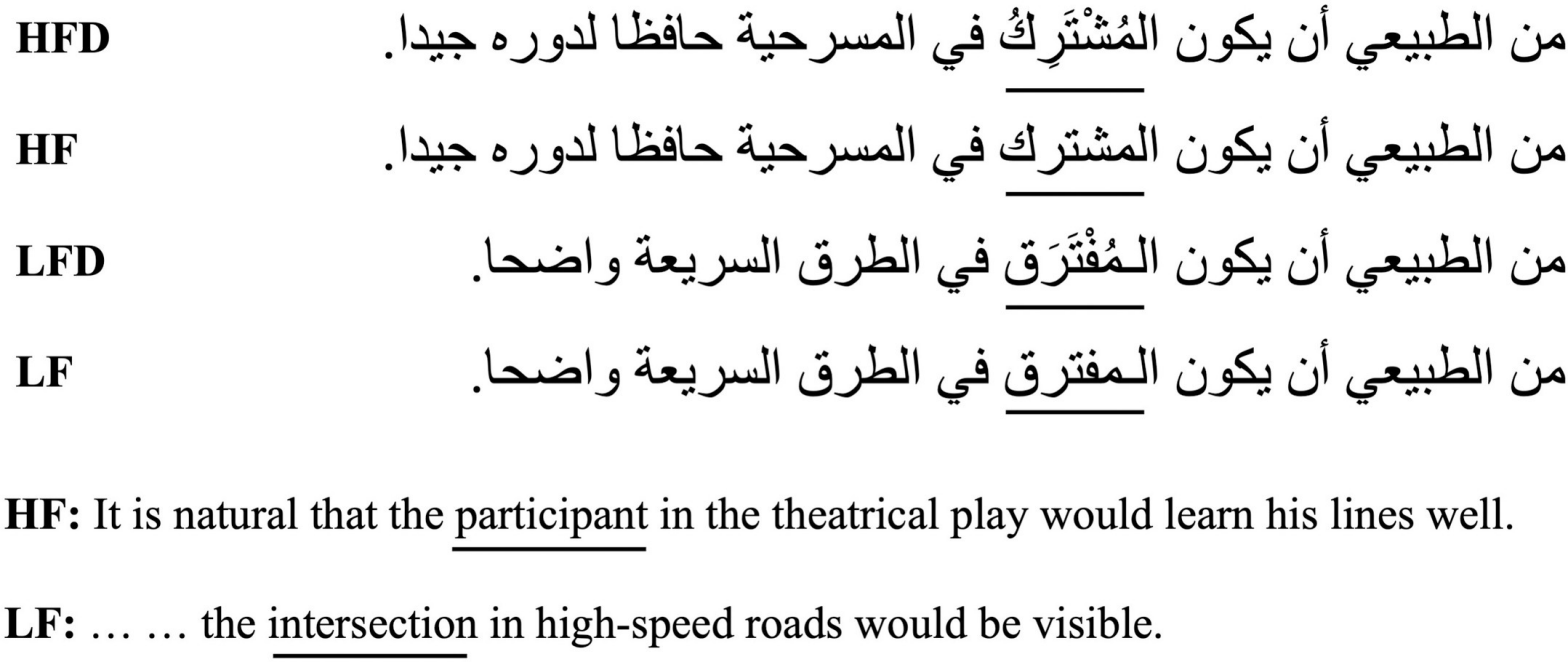
A sample stimuli set for Experiment 1. The target words are underlined in the Arabic frame sentences and the English translation. HF and LF are high- and low frequency target words conditions, respectively, and HFD and LFD are diacritized high- and low frequency target words conditions, respectively.

The undiacritized high- and low-frequency target word pairs were embedded in frame sentences that were identical up to the target word, with the pre-target context being non-constraining. Following the target word, the sentence context was allowed to vary to suit the meaning of the high- or low-frequency target word. Diacritics were added to the high- and low-frequency target word pairs in the same sentence frames to create the diacritized conditions. Thus, the diacritized and undiacritized high-frequency targets appeared in completely identical sentences, and the same applied to low-frequency words. The frame sentences contained on average 11 words (~ 63 characters, including spaces). The target word was always placed near the middle of the sentence. A sample stimuli set of the frequency × diacritization manipulation is provided in Fig 1.

*Experiment 2*: *Word length × diacritization stimuli*. Twenty-eight pairs of short (4-letter) and long (6-letter) words were used as target words. As in previous investigations of word length effects in Arabic and other languages (see above), the longer, 6-letter, words occupied wider spatial extent on the screen relative to the shorter 4-letter words (mean difference in spatial extent = 13.3 pixels, SD = 6.6, range = 4–26). The short and long words were matched on orthographic frequency (Aralex mean CPM _short words_ = 30.8, SD = 45.5; and mean CPM _long words_ = 26.4, SD = 0.83; *t*(54) < 1). Similarly, the two sets of words were also matched on age of acquisition (mean _short words_ = 9.7 years, SD = 0.9, range = 7.8–11.0; and mean _long words_ = 9.3 years, SD = 0.8, range = 7.6–11.0; *t*(54) = 1.7, *p* = 0.10). The short and long words were either undiacritized or with the diacritics that instantiated the subordinate pronunciation. For this experiment as well, the undiacritized and diacritized words (in both the short and long conditions) would instantiate the same pronunciation once placed in a sentence.

**Fig 2:**
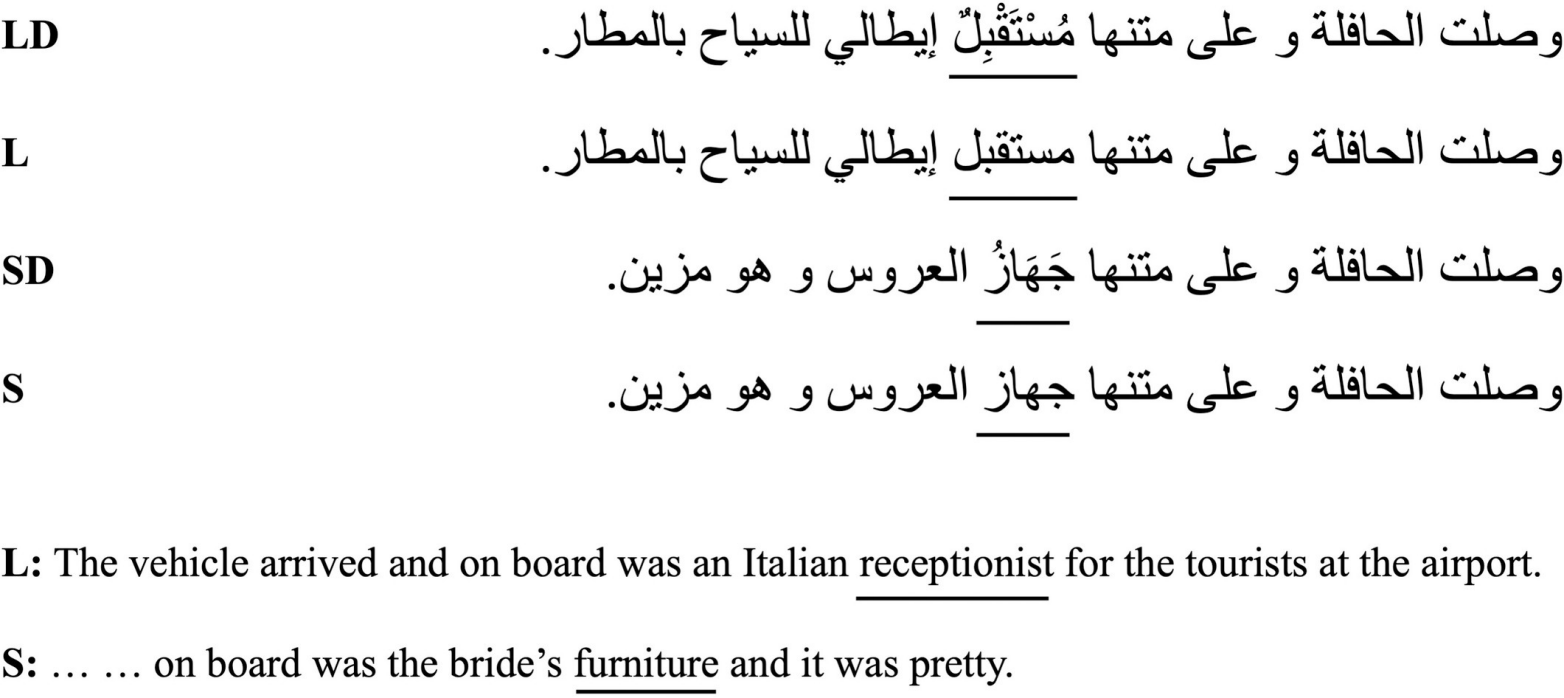
A sample stimuli set for Experiment 2. The target words are underlined in the Arabic frame sentences and the English translation. L and S are long (6 letter) and short (4 letter) target words conditions, respectively, and LD and SD are diacritized long and short target words conditions, respectively.

The undiacritized short and long target word pairs were embedded in frame sentences that were identical up to the target word, with the pre-target context being non-constraining. Following the target word, the sentence context was allowed to vary to suit the meaning of the short or long target word. Diacritics were added to the short and long target word pairs, and the diacritized words appeared in the same frame sentences that encompassed the undiacritized pairs. Thus, the diacritized and undiacritized short target words appeared in completely identical sentences, and the same applied to the long words. The frame sentences contain on average 10 words (~ 59 characters, including spaces). The target word was always placed near the middle of the sentence. A sample stimuli set of the length × diacritization manipulation is provided in Fig 2.

#### Norming procedure

For all stimuli of the three experiments, the following were the steps in which the norming was conducted. The first step was to establish the subordinate and dominant pronunciations of the potential target words. To this end, the participants who took part in the norming study were given a set of 256 undiacritized homographic words, and were asked to put each word in a complete and meaningful sentence. Only grammatically sound sentences were used to establish the pronunciation dominance of the ambiguous target words. A pronunciation of a particular word was deemed subordinate if it was instantiated ≤ 40% of the time in the produced sentences and an alternative pronunciation was produced more frequently. If more than one subordinate pronunciation was given by the participants, the one that was given least times was chosen to be used in the subsequent stages of norming. Only subordinate pronunciations were used in the subsequent norming stages. The participants were naïve as to the ultimate purpose of this activity.

The following stages aimed at establishing that these words are still in use and are known to typical Arabic readers (given that all the words conformed to the subordinate pronunciations). To this end, the participants were asked to indicate the correct definition of each word in a multiple-choice task (one of the options available was “I do not know this word”). The words used in the subsequent stages of norming were all known to the participants.

The following step was to establish the age of acquisition of the remaining words on the list. The participants supplied the estimates summarized above concerning the age they thought they acquired each word.

#### Design

A 2 word frequency (high, low) × 2 diacritization (diacritized, undiacritized) design was adopted in Experiment 1, with frequency and diacritization being the within-subject independent variables. The stimuli were counterbalanced using a Latin square and presented in pseudorandom order. Thus, participants saw each target only once, with equal number of high- and low-frequency words, diacritized and undiacritized in the testing session (i.e., 14 items per condition). The same 2 × 2 design, and counterbalancing and randomization procedures were adopted in Experiment 2, with word length (short, long) and diacritization (diacritized, undiacritized) being the two within-subject independent variables (also 14 items per condition).

#### Apparatus

The apparatus was identical for all three experiments. An SR Research Eyelink 1000 eye tracker was used to record participants’ eye movements during reading. Viewing was binocular, but eye movements were recorded from the right eye only. The eye tracker sampling rate was set at 1000 Hz. The eye tracker was interfaced with a Dell Precision 390 computer and with a 20-inch ViewSonic Professional Series P227f cathode ray tube (CRT) monitor (resolution 1024 × 768 pixels). A headrest was used to minimize participants’ head movements. The sentence text was displayed in black (Traditional Arabic font size 18, equivalent to the size of English print in Times New Roman font size 14) on a light grey background. Each sentence fitted in a single line. The display was 73 cm from the participants, and at this distance, on average, 2.3 characters equaled 1° of visual angle. The participants used a VPixx RESPONSEPixx VP-BB-1 button box to enter their responses to comprehension questions and to terminate trials after reading the sentences.

#### Procedure

The study was approved by the University of Southampton Ethics Committee. Data for both experiments were collected in the same session, with the sentences for each experiment acting as filler items for the other. The items of a third unrelated experiment were also presented to the participants in the same session, and acted as additional filler items. The experimental task was explained to the participants upon arrival at the lab and consenting participants began by taking part in Arabic reading proficiency screening tasks. These tasks consisted of reading aloud a printed paragraph (82 words), extracted from an Arabic newspaper, and also reading sentences aloud from the computer monitor. Only participants with 100% reading aloud accuracy rate were allowed to proceed to the actual eye tracking procedure.

The eye tracker was calibrated using a horizontal 3-point calibration at the beginning of the experiment, and the calibration was validated. Calibration accuracy was always ≤ 0.25°, otherwise calibration and validation were repeated. Prior to the onset of the target sentence, a circular fixation target (diameter = 1°) appeared on the screen in the location of the first character of the sentence, to the right side of the screen.

The participants were required to read silently, starting with ten practice sentences to become familiar with the procedure, before continuing on to the experimental sentences. The participants pressed a button once reading a sentence was finished, and this changed the display to the screen with a fixation target, and after this target was fixated the new sentence was displayed. On 25% of trials, pressing this button brought up a comprehension question to which the participants provided a yes/no answer using the same response box, prior to the onset of the screen with the fixation target. Participants were allowed to take as many breaks as they needed after which the eye tracker was re-calibrated and the calibration was validated. Testing sessions lasted approximately 45–60 minutes.

A final screening task to assess the participants’ proficiency in decoding diacritics accurately was performed after the eye tracking procedure. In this task participants were required to read aloud a list of 60 words, including 36 diacritized words. This task was conducted subsequent to the eye tracking procedure so that the participants were not alerted to the experimental interest in processing diacritics. Only eye movement data from highly proficient participants (diacritics decoding accuracy > 80%) were included in the reported analyses.

### Results

The sentence comprehension scores were analyzed separately for each of the experiments, and the results indicated that the participants were highly skilled. Experiment 1 (word frequency × diacritics) mean comprehension score = 91.1% (SD = 5.4, range = 78.1–100%); and Experiment 2 (word length × diacritics) mean comprehension score = 90.8% (SD = 5.7, range = 77.4–100%).

Launch distance is the distance between the location of the last pre-target fixation and the location of the first fixation on the target word. Existing evidence suggests that pre-processing of Arabic diacritics from a distant launch site may reduce the accuracy and efficiency of processing the diacritized target word, given the small size of diacritics relative to letters [32, 54]. A small percentage of trials where launch distance into the target word was > 4° (~ 9 characters) were removed from the analyses (1.1% in Experiment 1; and 0.9% in Experiment 2).

In both experiments, we report a number of eye movement measures for the target word region. These are (i) *word skipping probability* (the probability that the target word was not fixated during first pass reading); first pass reading measures, namely (ii) *first fixation duration* (the duration of the first fixation in first pass reading on the target word, regardless of the number of fixations the word received overall); (iii) *single fixation duration* (the duration of the fixation on the target in first pass reading in instances where the target received exactly one fixation during sentence reading); and (iv) *gaze duration* (the sum of fixation durations the target word received during first pass reading and before exiting the target word to go forward or backwards in the text). We also report (v) *go past time* (the sum of all fixation durations made from entering the target word region until the first fixation to the right of the target word. This measure includes regressions originating from the target word); (vi) *total fixation count* (the total number of fixations a word received from all passes); and (vii) *total fixation time* (the sum of all fixation durations the target received).

For the end of sentence region, we report the measure of *go past time* (the sum of fixation durations from the time of entering the end of the sentence region until the end of the trial, as there is no region further to the right of it), as discussed above. For this analysis, in both experiments, the contrast targeted diacritized vs. non-diacritized sentences, collapsing across the word frequency conditions (Exp. 1), and similarly collapsing across word length conditions (Exp. 2) conditions. This contrast was possible given that, with the exception of the presence or absence of the diacritics on the target, the diacritized and non-diacritized sentences were identical.

We used the *lme4* package (version 1.1–23 [55]) within the R environment for statistical computing [56] to analyze the raw fixation duration measures by fitting generalized linear mixed-effects models (GLMMs), with Gamma-distribution assumed for the fixation durations that were the dependent variables. The use of these GLMMs removes the need for the fixation duration measures to be normally distributed and as such there is also no need for prior transformation of the data [57]. For word skipping probability we used logistic GLMMs to account for the binary nature of this variable. We always started by running models with maximal random structure [58]. We trimmed the models when failure to converge, or when singular boundaries (a sign of overparameterization) were identified. Trimming the random effects structure was done first by removing interactions between random effects and then, if necessary, by also removing slopes. All findings reported here are thus from successfully converging models. This procedure was followed in analyzing the data in all three experiments.

### Experiment 1: Word frequency × diacritization

Prior to running the models, we prespecified the contrasts between the levels of the two fixed factors (target word frequency and diacritization, +.5/-.5 coding for each factor), using the contr.sdif function in the MASS package [59]. In all models, subjects and items were specified as the random variables.

For each of the eye movement measures, we report beta values (b), standard error (SE), *t* statistic for fixation durations and count measures, *z* statistic for skipping probability, and the *p* value associated with the *t* or *z* statistic. Furthermore, Bonferroni correction was applied to reduce family-wise error rate resulting from running multiple contrasts on the eye movement measures at the target word region [60]. For all target word analyses, the Bonferroni-corrected α = .05 ÷ 7 eye movement measures ≤ .007 was be adopted. For the analysis at the end of sentence region we only report one measure of eye movements and so α = .05 was adopted.

#### i. Target word analysis

The descriptive statistics for all reported eye movement measures at the target word region are listed in Table 1. Table 2 details the GLMM analyses output.

**Table 1:** Descriptive statistics of eye movement measures at target word region (experiment 1 –word frequency × diacritization).

	High Frequency		Low Frequency	
	Diacritized	Non-Diacritized	Diacritized	Non-Diacritized
	Mean (SD)	Mean (SD)	Mean (SD)	Mean (SD)
Skipping (probability)	0.04 (0.20)	0.06 (0.24)	0.05 (0.22)	0.06 (0.23)
First Fixation Duration (ms)	286 (125)	261 (94)	305 (128)	296 (122)
Single Fixation Duration (ms)	300 (11)	267 (6)	316 (10)	313 (9)
Gaze Duration (ms)	475 (303)	345 (188)	522 (355)	421 (250)
Go Past (ms)	562 (418)	424 (325)	633 (448)	475 (370)
Total Fixation Count	4.1 (3.6)	3.2 (2.4)	3.9 (3.1)	3.5 (2.9)
Total Fixation Time (ms)	1160 (1025)	819 (644)	1183 (960)	940 (826)

**Table 2:** GLMM output for eye movement measures (Experiment 1).

	Target Word Region			
	b	SE	*t* / *z*	*p*
	Skipping			
(Intercept)	-2.27	0.61	-3.69	**.0002**
Diacritized vs. Non-Diacritized	0.86	0.95	0.91	.3646
High vs. Low Frequency	-0.19	0.12	-1.53	.1250
Diacritization x Frequency	-0.13	0.20	-0.66	.5069
	First Fixation Duration			
(Intercept)	289.70	9.16	31.62	**< .0001**
Diacritized vs. Non-Diacritized	-16.36	5.78	-2.83	**.0047**
High vs. Low Frequency	26.60	5.78	4.60	**< .0001**
Diacritization x Frequency	10.66	10.07	1.06	.2899
	Single Fixation Duration			
(Intercept)	309.83	11.79	26.28	**< .0001**
Diacritized vs. Non-Diacritized	-18.76	6.99	-2.69	.0072
High vs. Low Frequency	33.59	7.06	4.76	**< .0001**
Diacritization x Frequency	21.44	12.26	1.75	.0803
	Gaze Duration			
(Intercept)	449.32	19.70	22.80	**< .0001**
Diacritized vs. Non-Diacritized	-98.81	9.72	-10.16	**< .0001**
High vs. Low Frequency	33.07	9.84	3.36	**.0008**
Diacritization x Frequency	24.53	15.32	1.60	.1094
	Go Past			
(Intercept)	541.66	12.82	42.24	**< .0001**
Diacritized vs. Non-Diacritized	-160.88	12.56	-12.81	**< .0001**
High vs. Low Frequency	74.19	15.93	4.66	**< .0001**
Diacritization x Frequency	-18.64	17.30	-1.08	.2810
	Total Fixation Count			
(Intercept)	3.64	0.37	9.74	**< .0001**
Diacritized vs. Non-Diacritized	-0.65	0.12	-5.60	**< .0001**
High vs. Low Frequency	0.12	0.12	0.99	.3200
Diacritization x Frequency	0.46	0.23	1.97	.0495
	Total Fixation Time			
(Intercept)	1028.85	20.84	49.37	**< .0001**
Diacritized vs. Non-Diacritized	-280.10	17.99	-15.57	**< .0001**
High vs. Low Frequency	81.68	18.98	4.30	**< .0001**
Diacritization x Frequency	55.94	22.07	2.53	.0113
	End of Sentence Region			
	b	SE	*t*	*p*
	Go Past			
(Intercept)	3946.32	11.49	343.32	**< .0001**
Diacritized vs. Non-Diacritized	50.32	13.42	3.75	**.0002**

Significant *p* values (Bonferroni-correct for target word measures) are marked in boldface. The final models that yielded these results are reported in S1 File.

*Skipping*. There was no significant main effect or interactions of word frequency and diacritization on the probability of word skipping.

*First pass reading measures*. The pattern of results obtained for first and single fixation, and gaze duration was almost identical. In all three measures there was a significant main effect of word frequency, in the expected direction, with shorter fixation durations on high-frequency target words. There was also a significant main effect of diacritization such that diacritized words attracted longer fixation durations compared to undiacritized words (in single fixation duration the effect (*p* = .0072) almost reached the Bonferroni-corrected alpha level *p* = .007). No significant interaction between word frequency and diacritization was found in any of the first pass reading measures.

*Go past time*. Similar to first pass reading measures, there was a significant effect for both word frequency and diacritization, in the same directions, and no significant interaction.

*Total fixation count*. Only a significant effect of diacritization was obtained such that diacritized words attracted more fixations compared to undiacritized words. There was no significant main effect of frequency. The interaction between frequency and diacritization did not survive the Bonferroni correction of the α value.

*Total fixation time*. Similar to first pass reading measures and go past, there was a significant effect for both word frequency and diacritization, in the same directions. The interaction between frequency and diacritization did not survive the Bonferroni correction for multiple comparisons.

*Bayesian analysis of interactions*. Given the absence of significant interactions between diacritization and word frequency effects, Bayesian analyses were conducted to quantify the amount of evidence the data provide for either the null hypothesis or the alternative hypothesis. We carried out the analysis by comparing two models. In both models, participants and items were specified as random factors. The first model did not feature an interaction between the fixed factors of word frequency and diacritization, whereas the second model did. The analyses were carried out using the BayesFactor package in the R environment (version 0.9.12–4.2, [61]) and used the default scale value (0.5) for the Cauchy priors on effect size, and 100,000 Monte Carlo iterations. BayesFactor values of <1 is considered to indicate evidence for the model without fixed factors interaction (i.e., evidence for the null hypothesis H_0_). Conversely, BayesFactor vales of >1are considered evidence for the model with fixed factors interaction (i.e., evidence for the alternative hypothesis H_1_). The BayesFactors values obtained from the analyses were: 0.09 for skipping (strong evidence for H_0_), 0.20 for first fixation duration (substantial evidence for H_0_), 0.43 for single fixation duration (anecdotal evidence for H_0_), 0.16 for gaze duration (substantial evidence for H_0_), 0.09 for go past time (strong evidence for H_0_), 0.60 for total fixation count (anecdotal evidence for H_0_), and 0.33 for total fixation time (substantial evidence for H_0_). The parenthetical descriptors are based on the categorization commonly used to interpret BayesFactor values, where values <1/3 constitute substantial evidence for the null effect, and <1/10 strong evidence [62, 63].

#### ii. End of sentence region analysis

*Go past time*. Go past time was significantly longer at the end of the sentences in the undiacritized condition (Mean = 3708, SD = 3979) relative to when the target words were diacritized (Mean = 3668, SD = 3406, see Table 2 for GLMM analysis output).

### Experiment 2: Word length × diacritization

#### i. Target word analysis

The descriptive statistics for all reported eye movement measures at the target word region are listed in Table 3. Table 4 details the GLMM analyses output.

**Table 3:** Descriptive statistics of eye movement measures (experiment 2 –word length × diacritization).

	Long Words		Short Words	
	Diacritized	Non-Diacritized	Diacritized	Non-Diacritized
	Mean (SD)	Mean (SD)	Mean (SD)	Mean (SD)
Skipping (probability)	0.03 (0.17)	0.02 (0.14)	0.05 (0.22)	0.06 (0.24)
First Fixation Duration (ms)	302 (130)	280 (113)	286 (125)	285 (118)
Single Fixation Duration (ms)	306 (132)	290 (121)	289 (126)	293 (117)
Gaze Duration (ms)	506 (314)	405 (241)	441 (319)	357 (179)
Go Past (ms)	639 (433)	544 (458)	595 (487)	424 (306)
Total Fixation Count	3.8 (3.0)	3.7 (2.7)	3.5 (2.7)	3.1 (2.4)
Total Fixation Time (ms)	1136 (909)	998 (779)	1020 (885)	849 (734)

**Table 4:** GLMM output for eye movement measures (experiment 2 –word length × diacritization).

	Target Word Region			
	b	SE	*t* / *z*	*p*
	Skipping			
(Intercept)	-3.40	0.22	-15.66	**< .0001**
Diacritized vs. Non-Diacritized	-0.12	0.32	-0.38	.7024
Long vs. Short Words	-0.91	0.32	-2.82	**.0048**
Diacritization x Length	-0.60	0.64	-0.93	.3550
	First Fixation Duration			
(Intercept)	290.43	8.89	32.66	**< .0001**
Diacritized vs. Non-Diacritized	-10.54	5.71	-1.85	.0649
Long vs. Short Words	5.66	5.65	1.00	.3160
Diacritization x Length	-19.40	10.25	-1.89	.0585
	Single Fixation Duration			
(Intercept)	299.02	10.31	29.02	**< .0001**
Diacritized vs. Non-Diacritized	-9.57	7.75	-1.24	.2170
Long vs. Short Words	7.55	7.75	0.98	.3290
Diacritization x Length	-20.16	14.58	-1.38	.1670
	Gaze Duration			
(Intercept)	431.09	17.89	24.10	**< .0001**
Diacritized vs. Non-Diacritized	-71.32	11.75	-6.07	**< .0001**
Long vs. Short Words	50.03	11.02	4.54	**< .0001**
Diacritization x Length	-29.94	16.08	-1.86	.0627
	Go Past			
(Intercept)	558.21	22.01	25.37	**< .0001**
Diacritized vs. Non-Diacritized	-100.84	11.30	-8.93	**< .0001**
Long vs. Short Words	53.73	14.62	3.67	**.0002**
Diacritization x Length	8.79	18.30	0.48	.6309
	Total Fixation Count			
(Intercept)	3.52	0.31	11.37	**< .0001**
Diacritized vs. Non-Diacritized	-0.28	0.11	-2.44	.0149
Long vs. Short Words	0.51	0.11	4.44	**< .0001**
Diacritization x Length	0.32	0.23	1.41	.1582
	Total Fixation Time			
(Intercept)	1052.84	17.28	60.93	**< .0001**
Diacritized vs. Non-Diacritized	-162.13	20.82	-7.79	**< .0001**
Long vs. Short Words	134.41	15.63	8.60	**< .0001**
Diacritization x Length	21.37	17.37	1.23	.2190
	End of Sentence Region			
	b	SE	*t*	*p*
	Go Past			
(Intercept)	3409.16	13.12	259.78	**< .0001**
Diacritized vs. Non-Diacritized	108.42	19.31	5.62	**< .0001**

Significant *p* values (Bonferroni-correct for target word measures) are marked in boldface. The final models that yielded these results are reported in S1 File.

*Skipping*. There was a significant main effect of word length on skipping probability, in the expected direction with shorter words being skipped more often than longer words. There was however no significant main effect of diacritization, and no interaction.

*First pass reading measures*. In first and single fixation durations, there were no significant main effects of word length or diacritization, nor significant interactions. In gaze duration, however, there was a significant main effect of word length, in the expected direction, and a significant main effect of diacritization such that diacritized words attracted longer fixation durations compared to undiacritized words. Similar to first and single fixation durations, there was no significant interaction between word length and diacritization in gaze duration.

*Go past time*. Similar to gaze duration, there was a significant effect for both word frequency and diacritization, in the same directions, and no significant interaction.

*Total fixation count*. Only a significant effect of word length was obtained such that longer words attracted more fixations than shorter words. There was no significant main effect of diacritization and no interaction between word length and diacritization.

*Total fixation time*. Similar to the gaze duration and go past measures, there was a significant effect for both word length and diacritization, in the same directions. There was no significant interaction between word length and diacritization.

*Bayesian analysis of interactions*. Similar to Exp. 1, Bayesian analyses were conducted to quantify the amount of evidence the data provide for either the null hypothesis or the alternative hypothesis. We used the same procedure of comparing models without and with interaction of the fixed factors. The BayesFactors values obtained from the analyses were: 0.13 for skipping (substantial evidence for H_0_), 0.29 for first fixation duration (substantial evidence for H_0_), 0.22 for single fixation duration (substantial evidence for H_0_), 0.11 for gaze duration (substantial evidence for H_0_), 0.38 for go past time (anecdotal evidence for H_0_), 0.19 for total fixation count (substantial evidence for H_0_), and 0.09 for total fixation time (strong evidence for H_0_).

#### ii. End of sentence region analysis

*Go past time*. Similar to the findings in Experiment 1, go past time was significantly longer at the end of the sentences in the undiacritized condition (Mean = 3298, SD = 3246) relative to when the target words were diacritized (Mean = 3188, SD = 3192, see Table 4 for GLMM analysis output).

### Discussion

The results from both experiments were largely consistent. To begin with, we obtained the expected classic word frequency effects in all first pass processing measures, and in go past time and total fixation time, with longer fixation times for low frequency compared to high frequency words. We also replicated word length effects in gaze duration, and in measures of later processing (go past time. total fixation count, and total fixation time), with longer words receiving longer fixation times than shorter words. Importantly, in Experiment 1, the effect of adding disambiguating diacritics that instantiated the subordinate analysis of the target words resulted in longer fixation durations on the target during almost all first pass reading measures and go past time, total fixation time, as well as more fixations on the target, relative to when the ambiguous target was undiacritized. This applied to both high- and low-frequency words, with no significant interaction between the variables of word diacritization and frequency. Similarly, in Experiment 2, diacritized targets attracted longer gaze duration, go past time and total fixation time relative to undiacritized targets. There was also no significant interaction between word diacritization and length.

In the light of the results from these two experiments, we can rule out that spotting the diacritics parafoveally has resulted in processing facilitation (additional evidence from pre-target word analyses are reported in S1 File). We will test this prediction once again in Experiment 3. The results suggest that the costs associated with the diacritics instantiating the subordinate phono-semantic representations of the ambiguous heterophonic homographs, and suppressing the dominant representations (i.e., the subordinate bias effect), affected the processing of these words regardless of their frequency, or length. Furthermore, we found no evidence that the presence of the disambiguating diacritics on the target word modulated word frequency and length effects. Indeed, in the both experiments there were no significant interactions between diacritization and the variables of word frequency and length, and the Bayesian analyses yielded evidence only for this outcome.

Downstream from the target words, at the end of the sentence region, the pattern of results was reversed. In both experiments the diacritized target word conditions yielded significantly shorter re-reading time, indexed by go past measure, relative to when the targets were undiacritized. This pattern suggests that readers made use of the diacritics when present on the target to disambiguate it, and as the remainder of the sentence confirmed the representation they adopted (the subordinate analysis of the target), reading progressed smoothly. By contrast, in the absence of the disambiguating diacritics on the target, the readers arguably adopted the dominant analysis of the homograph. This allowed them to progress through the target word region with relative ease, with shorter first pass and re-reading time compared to when the target was disambiguated by the diacritics. As the rest of the sentence instantiated the subordinate representation of the target, however, the readers’ analysis of the sentence including the target was challenged, resulting in substantial increase in re-reading at the end of sentence region. These findings replicate previous reported results [e.g., 34, 36, 40, 42]. Further discussion of the implications of these results will follow in the General Discussion.

## Experiment 3: Word predictability and diacritics-based disambiguation

Whereas word frequency and length effects pertain to word-level properties and processes, word predictability effect indexes the extent to which sentence context facilitates the identification of a predictable word (e.g., [10, 20–22, 27]). In the current experiment, we aimed to replicate word predictability effects in Arabic homographic target words, as well as explore the potential interplay between diacritics-based disambiguation and predictability.

In the case of ambiguous homographic words, placing such words after context that guides the reader to predict a particular word arguably resolves the bulk, if not all, of the ambiguity and makes the use of diacritics superfluous. As such, we were constrained to use diacritics only with low-predictability targets, where the use of diacritics would be deemed ecologically valid, that is, where the previous context does not guide the readers to adopt one particular representation of the homograph or make it predictable. Consequently, we investigated the classic predictability effects by contrasting high- and low-predictability conditions, and examined the effects of diacritization of low-predictability words by contrasting diacritized and undiacritized low-predictability targets. The subordinate representation of the target homographs was always instantiated (by diacritics or the subsequent context).

If contextually predictable words are easier to identify because previous context has already activated some aspects of their representations (e.g., semantic, syntactic, or phonological, see e.g. [21]), then it is plausible that in the absence of contextual predictability, another source that provides additional information about a word’s pronunciation and meaning may facilitate its identification. Arabic diacritics, as discussed above, are such an additional source of information that would serve to fully disambiguate the phono-semantic representation of the ambiguous word. Additionally, and as discussed above, spotting the diacritics parafoveally, prior to fixating the target, may allow readers to expect and adopt the subordinate phono-semantic representation of this word. This spot-activate-verify mechanism may thus offset, even to a small extent, the processing costs of the target word being of low predictability in the context in which it is embedded. Thus, the current experiment perhaps provides the ultimate test of this hypothesis, with the diacritics allowing the target’s phono-semantic representation to become expected and activated prior to fixating it potentially reducing the cost of the target not being predictable from preceding context. If diacritized low-predictability words become faster to identify relative to when undiacritized, we may conclude that diacritics-based disambiguation attenuated (low) predictability effects.

However, another plausible scenario would be that the presence of diacritics that instantiate the subordinate representation of the homographic words results in added processing costs as a manifestation of the subordinate bias effect (see above, e.g., [34, 35]), as was observed in Experiments 1 and 2. If this is the case, then the diacritization will compound the difficulty of processing of the low-predictability targets.

As with Experiments 1 and 2, we also examined whether diacritizing the target word facilitated sentence processing by reporting readers’ re-reading activity at the end of sentence region (go past measure). In this respect, we forward the same hypotheses outlined in Experiments 1 and 2. Namely, as the subordinate analysis of the target is instantiated by the disambiguating diacritics, and the rest of the sentence confirms this analysis, no disruption in later sentence processing would be observed. Whereas, if in the absence of diacritics readers fail to suppress the dominant representation of the homographic target, their analysis will be challenged by the subsequent sentence context, and disruption will be observed at later integrative sentence processes.

### Method

*Participants*. Thirty-six native Arabic speakers (17 women; mean age = 30.8 years, SD = 9.0, range = 20–65) participated in the eye tracking procedure after giving written informed consent.

*Stimuli*. Thirty pairs of high- and low predictability words were used as targets. As with the frequency and length stimuli, the high- and low-predictability target words were the subordinate versions of common Arabic heterophonic-homographs. The high- and low-predictability words were matched on orthographic frequency (Aralex mean CPM _high-predictability_ = 46.7, SD = 74.4; and mean CPM _low-predictability_ = 64.7, SD = 132.1; *t*(58) < 1). Similarly, the two sets of words were also matched on age of acquisition (mean _high-predictability_ = 8.5 years, SD = 1.2, range = 7–10.2; and mean _low-predictability_ = 8.9 years, SD = 1.0, range = 7–10.8; *t*(58) < 1). The high- and low-predictability word sets were matched on word length (for both sets, mean = 4.7 characters, SD = 1.2, range = 3–6) and on spatial extent.

**Fig 3:**
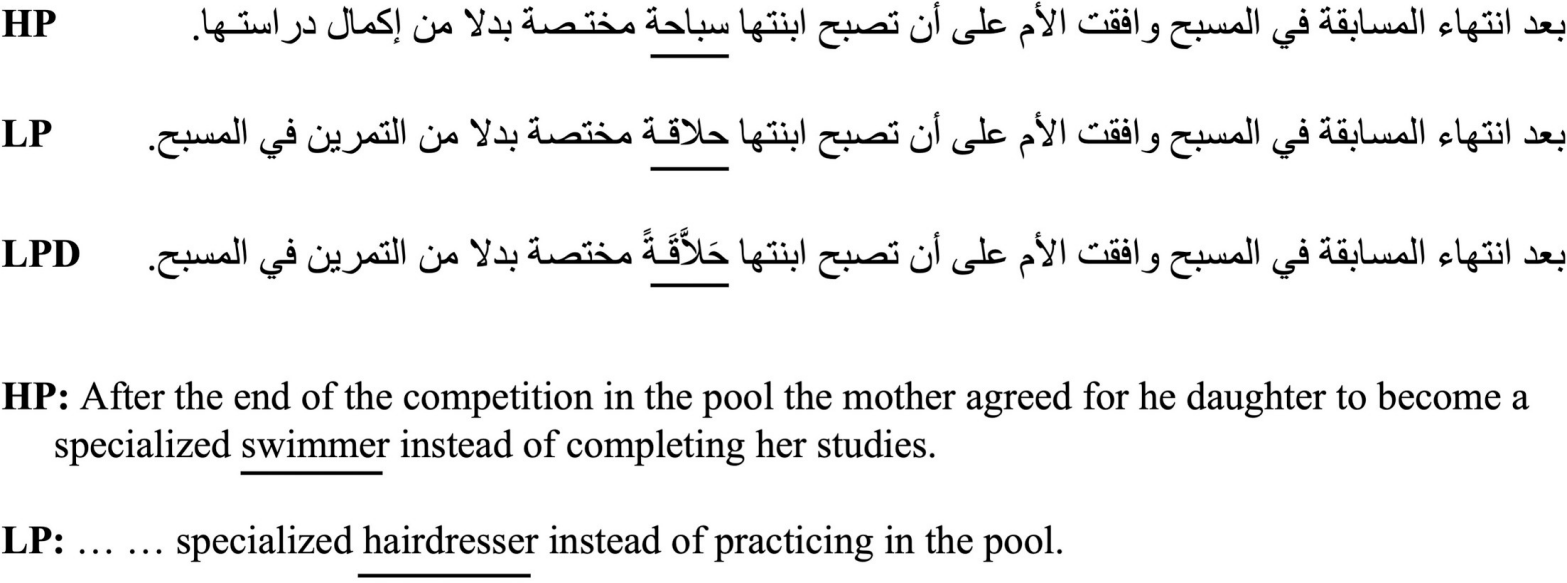
A sample stimuli set for Experiment 3. The target words are underlined in the Arabic frame sentences and the English translation. HP and LP are high- and low-predictability target words conditions, respectively, and LPD is the diacritized low-predictability condition.

The undiacritized high- and low-predictability target word pairs were embedded in frame sentences that were identical until the target word. Subsequent to the target word, the sentence context was allowed to vary to suit the high- or low-predictability targets. Diacritics were added to the low-predictability words thus creating the diacritized low predictability condition, and the diacritized targets appeared in the same frame sentences that encompassed the undiacritized targets. Thus, the diacritized and undiacritized low-predictability targets appeared in completely identical sentences. The frame sentences contained on average 15 words (~ 81 characters, including spaces). The target word was always placed near the middle of the sentence. A sample stimuli set for the predictability and diacritization manipulation is provided in Fig 3.

*Norming procedure*. In addition to the norming steps listed above (Experiments 1 and 2) to establish meaning dominance, familiarity with the target words etc., the target words intended for the high- and low-predictability conditions were selected using a cloze task. The words in the high-predictability condition were produced 100% of the time (i.e., by all 12 participants who took part in this task), whereas the low-predictability words were never produced (i.e., produced by 0 participants).

*Design*. The effects of word predictability and diacritization were assessed separately through adopting three within-subject one-way experimental conditions: high-predictability (undiacritized), low-predictability (undiacritized), and low-predictability (diacritized). The stimuli were presented in random order and counterbalanced such that an equal number of stimuli from each condition was presented, and each presented item appeared only once in the testing session. The apparatus and experimental procedure were identical to Experiments 1 and 2. Notably, items from another unrelated experiment were used as filler items for the target sentences of the current experiment.

### Results

The sentence comprehension scores indicated that the participants were reading for comprehension: mean score = 90.2% (SD = 6.1, range = 83.3–100%).

The analyses reported below used the data points of only 26 items of the stimuli set, with 4 items excluded from the analyses upon discovering errors in sentence structures of these items. In the remaining data set, as with Experiments 1 and 2, a small percentage (0.6%) of trials where launch distance into the target word was > 4° (~ 9 characters) were removed from the analyses.

We report the same eye movement measures on the target word as in Experiments 1 and 2. We also report the go past measure at the end of sentence region for the diacritized vs. undiacritized low-predictability conditions. The inferential analyses were also run in a similar manner to Experiments 1 and 2, including the Bonferroni correction to reduce family-wise error rate resulting from running multiple contrasts in the target word region. Specifically, sliding contrasts were prespecified using the contr.sdif function in the MASS library to reveal predictability effects (high- vs. low-predictability conditions), and to reveal diacritization effects (low-predictability vs. low predictability diacritized conditions). Model trimming was performed as described above when necessary (e.g., when singular fit was identified). In the case of the measure of skipping, not even intercept only models converged. The only GLMM that converged and did not result in a singular fit contained items only intercept.

#### i. Target word analysis

The descriptive statistics for all reported eye movement measures at the target word region are listed in Table 5. Table 6 details the GLMM analyses output.

**Table 5:** Descriptive statistics of eye movement measures (experiment 3 –word predictability and diacritization).

	High Predictability	Low Predictability	Low Predictability Diacritized
	Mean (SD)	Mean (SD)	Mean (SD)
Skipping (probability)	0.20 (0.40)	0.27 (0.45)	0.17 (0.38)
First Fixation Duration (ms)	264 (102)	285 (109)	301 (135)
Single Fixation Duration (ms)	275 (102)	296 (116)	318 (126)
Gaze Duration (ms)	351 (185)	402 (221)	463 (293)
Go Past (ms)	422 (320)	554 (750)	568 (438)
Total Fixation Count	2.2 (1.5)	2.9 (2.0)	3.0 (2.0)
Total Fixation Time (ms)	587 (456)	791 (599)	884 (662)

**Table 6:** GLMM output for eye movement measures (experiment 3 –word predictability and diacritization).

	Target Word Region			
	b	SE	*t* / *z*	*p*
	Skipping			
(Intercept)	-2.31	0.50	-4.64	**< .0001**
High vs. Low Predictability	0.77	0.26	2.98	**.0029**
Low Predictability vs. Low Predictability Diacritized	-1.05	0.27	-3.95	**.0001**
	First Fixation Duration			
(Intercept)	282.82	11.27	25.10	**< .0001**
High vs. Low Predictability	22.55	11.07	2.04	.0416
Low Predictability vs. Low Predictability Diacritized	10.98	12.08	0.91	.3635
	Single Fixation Duration			
(Intercept)	299.46	12.23	24.48	**< .0001**
High vs. Low Predictability	26.12	16.31	1.60	.1090
Low Predictability vs. Low Predictability Diacritized	18.13	18.80	0.96	.3350
	Gaze Duration			
(Intercept)	407.66	19.64	20.76	**< .0001**
High vs. Low Predictability	29.19	13.38	2.18	.0291
Low Predictability vs. Low Predictability Diacritized	43.42	14.30	3.04	**.0024**
	Go Past			
(Intercept)	519.01	20.43	25.40	**< .0001**
High vs. Low Predictability	45.64	15.46	2.95	**.0032**
Low Predictability vs. Low Predictability Diacritized	37.21	17.13	2.17	.0298
	Total Fixation Count			
(Intercept)	2.69	0.21	12.99	**< .0001**
High vs. Low Predictability	0.66	0.13	5.26	**< .0001**
Low Predictability vs. Low Predictability Diacritized	0.09	0.13	0.71	0.4760
	Total Fixation Time			
(Intercept)	792.53	22.94	34.55	**< .0001**
High vs. Low Predictability	147.66	16.76	8.81	**< .0001**
Low Predictability vs. Low Predictability Diacritized	120.00	18.71	6.42	**< .0001**
	End of Sentence Region			
	b	SE	*t*	*p*
	Go Past			
(Intercept)	3795.99	30.45	124.68	**< .0001**
Low Predictability vs. Low Predictability Diacritized	-122.94	22.83	-5.38	**< .0001**

Significant *p* values (Bonferroni-correct for target word measures) are marked in boldface. The final models that yielded these results are reported in S1 File.

*Predictability effects*. The well-documented word predictability effects were obtained in skipping, first fixation and gaze durations, go past time, total fixation count, and total fixation time. However, the effect survived the Bonferroni correction for multiple testing in go past time, total fixation count, and total fixation time.

*Diacritization effects*. The presence of diacritics on the low-predictability target words resulted in significantly reduced skipping probability. Additionally, diacritization also resulted in increased reading times in gaze duration, go past time and total fixation time. The pattern of fixation duration results strongly resembles the effects of diacritization reported in Experiment 2. The effect survived the Bonferroni correction in measures of skipping, gaze duration, and total fixation time.

#### ii. End of sentence region analysis

*Go past time*. Similar to the findings in Experiments 1 and 2, go past time was significantly longer at the end of the sentences in the undiacritized condition (Mean = 3708 SD = 3580) relative to when the target words were diacritized (Mean = 3438, SD = 3479, see Table 6 for GLMM analysis output).

#### Discussion

The data trends reported are in line with the word predictability effect. For instance, early processing and first pass measures showed that low-predictability targets resulted in 7% reduction in skipping rate, and attracted on average 21 ms longer first fixation durations, 51 ms longer gaze durations. Predictability effects were also obtained in later processing measures with low predictability targets attracting 132 ms longer go past time, and 204 ms longer total fixation time, relative to high-predictability words, in addition to the significant increase in total fixation count for low-predictability words.

With regards to the effects of the diacritics-based disambiguation, the results largely replicated the findings from Experiments 1 and 2. The presence of these disambiguating diacritics on the low-predictability targets did not speed up their identification. Rather, diacritization resulted in significant reduction in skipping rates (10%), as well as significantly increased gaze duration, a marginal increase in go past time, and a substantial increase in total fixation time. We, thus, have no evidence that the information supplied by the diacritics compensated for the low-predictability status of the diacritized targets, and, once again, no evidence that spotting diacritics parafoveally facilitated the processing of the disambiguated homograph once it was fixated (again, note that additional evidence from pre-target word analyses are reported in S1 File).

Also similar to what was reported in Experiments 1 and 2, at the end of the sentence region, the pattern of results was reversed as the diacritized target word condition yielded significantly shorter go past measure, relative to the undiacritized condition. This pattern suggests that readers made use of the diacritics on the target, and that the remainder of the sentence confirmed the subordinate representation of the homograph that was instantiated by the diacritics. Whereas in the absence of the disambiguating diacritics the readers must have adopted the dominant representation of the target, only to have this representation challenged later on in subsequent sentence regions, resulting in a significant increase in processing time (re-reading).

## General discussion

The reported experiments replicated the basic word frequency, length and predictability effects in Arabic. In addition, the results were informative with regards to exploratory questions that motivated this research, namely, whether the effects of diacritics-based disambiguation during sentence reading would modulate word frequency, length and predictability effects. In Experiments 1 and 2 we did not find evidence that diacritics-based disambiguation modulated the effect of word frequency or length: There were no statistically reliable interactions between diacritization and these effects. The presence of diacritics increased readers’ early (first pass) processing time, and also the attempts to integrate the diacritized target with prior context (go past measure on the target words), as well as in total fixation time on the diacritized targets. This was the case for both high- and low-frequency words (Experiment 1), and long and short words (Experiment 2). The processing costs observed on diacritized targets did not differentially affect words in the harder-to-process conditions (e.g., low-frequency or longer words).

Similarly, in Experiment 3, adding disambiguating diacritics to the low-predictability ambiguous targets did not facilitate the identification of these words, relative to when the diacritics were absent. Rather, there was a significant reduction in skipping rates, and a similar pattern of increased processing time on the diacritized target words. The idea that adding the diacritics would, at least to some extent, speed up the identification of words that are not predictable from previous context was not supported by our findings. Similar to Experiments 1 and 2, there was no evidence that spotting the diacritics parafoveally and activating the subordinate phono-semantic representation of the homographic target facilitated the processing of this target once fixated. Rather, the reduction in skipping rate of diacritized words replicated previous findings [38], suggesting that readers may adopt a more cautious processing strategy (e.g., reduced skipping) in respect of an upcoming diacritized word.

In all three experiments, the inflated processing time on the diacritized target words most likely reflect the costs associated with (a) the processing of the additional phono-semantic information supplied by the diacritics, and (b) the homograph disambiguation processes that includes activating the subordinate representation, and suppressing the more readily accessible dominant representation (i.e., the subordinate bias effect). Thus, this is the first time, to our knowledge, the subordinate bias effect was obtained by instantiating the subordinate representation via characteristics of the homographic word itself rather than through manipulation of the characteristics of the prior context, as was consistently the case in the previous studies reviewed above.

As discussed above, we are not aware of a theoretical framework that would predict that diacritics-based disambiguation would have affected easier-to-process words (i.e., high-frequency and short words, Exps. 1 and 2) differently than their harder-to-process counterparts, that is an interaction between diacritization (i.e., disambiguation) and the variables of word frequency and length. As discussed above, in biased homographs, such as the targets in all reported experiments, the subordinate representation, or representations, occur in the language less frequently than the dominant representation. As such, these subordinate representations that are instantiated by the diacritics are, by definition, low-frequency words. In effect, instantiating the subordinate representations turned all target words into (even) lower-frequency versions, and hence produced the processing costs that were reported in all diacritized conditions, in all experiments, and with no interaction with the variables of word frequency and length in Experiments 1 and 2. It is worth noting however, that previous investigations revealed some differences between processing of low-frequency unambiguous words, and ambiguous words that were disambiguated such that a low-frequency (subordinate) representation was instantiated. For instance, Sereno et al., [33] found that although the patterns of eye movements on both types of words were similar, the disambiguated words attracted more regressions. In a later investigation, Sereno et al., [48] reported a step-like function: Fixation durations on the disambiguated homographs (instantiating the subordinate representation) fell between shorter fixation durations on higher-frequency controls that matched the frequency of the overall word form of the ambiguous homographs, and the much longer fixation durations on low-frequency controls that matched the frequency of the subordinate representations of the homograph. The limited availability of databases that list the frequency counts of subordinate representations of Arabic homographs prevented us from utilizing this type of frequency matching. Given the linguistic properties of Arabic (e.g., the abundance of homographic words), it can be a fertile linguistic environment to further investigate the subordinate bias effect and to what extent it overlaps or diverges from word or meaning frequency effects. The theoretical contributions of such research would be considerable (see e.g., [35]).

Instantiating the subordinate representation on the target itself through the diacritics facilitated later processing of the sentences. Specifically, integrating the diacritized target word into the overall sentence representation was easier as both the diacritics and the subsequent context instantiated the subordinate representation of the targets. By contrast, in the absence of the disambiguating diacritics on the targets, readers’ processing of the sentence was marked by disruption and lengthier integration processes. This manifested as a significant inflation of go past time on the end of sentence region, compared to when the targets were diacritized. This indicates that in the absence of diacritics, readers adopted the dominant representation of the target, and this analysis was challenged in subsequent sentence regions that instantiated the subordinate representation of the targets.

Given the dominance of heavily biased homographs in Arabic, which is reflected in the stimuli selection, the inclusion of contrast conditions such as balanced homographs (diacritized or not) was not possible. As such, our results cannot really be used to evaluate models that posit that in the absence of constraining or disambiguating context, the competition between the different representations of these homographs influences the processing time required (e.g., the reordered access model, see [50, 61] for reviews). This competition was kept minimal in all reported experiments. Similarly, given that we could not include control conditions where diacritized homographs followed disambiguating context, to ensure that the use of the diacritics was ecologically valid, the reported results cannot be used to adjudicate between modular versus integrative accounts of lexical ambiguity resolution. Modular accounts (also referred to as *autonomous access models*, e.g., the integration model [37]), mainly rule out any role of context in selecting the representation of the homograph that should be accessed. By contrast, integrative models (also referred to as *selective access models*, e.g., the reordered access model, [64]; see [50] for review) postulate that context may play some (or even a major) role in selecting a particular representation of the homographs.

All that said, the patterns of results we obtained may perhaps lend some additional support to the remaining aspects of the reordered access model. This model remains the only theoretical (and computationally implemented) framework that successfully accommodates the subordinate bias effect [50]. Specifically, if we adopt the plausible interpretation that the inflated processing time on the diacritized targets in all experiments is a replication of the subordinate bias effect (given that the diacritics instantiated the subordinate representations of these targets), the following conclusions are possible. In line with the reordered access model assumptions [64], both dominant and subordinate representations of the target homographs must have become available to the readers simultaneously. In the absence of the disambiguating diacritics, the dominant representation was adopted with minimal competition. By contrast, when the diacritics that instantiated the subordinate representation were present, the readers had to suppress the easily accessible dominant representation, hence the inflated processing time on the diacritized targets. Furthermore, and also in line with the predictions of the reordered access model, the disruption to processing observed downstream at the end of sentence region, for the undiacritized target conditions in all experiments, unequivocally supports the idea that when readers encounter biased homographs that are not disambiguated by context (or by diacritics, in the case of Arabic), the readers adopt the dominant analysis. This analysis was however challenged as the post-target sentence context instantiated the subordinate representation of the homographs. Notably, this end of sentence disruption to processing was not observed when the readers encountered the disambiguating diacritics on the target.

The idea that readers adopt the dominant representation in the absence of diacritics and prior constraining context is perhaps also in line with the principles of the Bayesian Reader model [65]. This model postulates that the word identification system functions optimally and readers are ideal observers. As such, it is plausible that the reader considers the prior probability of the word occurrence, and hence words that occur more frequently are easier to identify (i.e., the word frequency effect, see e.g., [66, 67]). Specifically, the probability *P* of observing the perceptual input *I*, given that the word *W* has been presented, is captured by the term *P*(*I* | *W*), and continuously updating the probability with each new encounter. It is possible to extrapolate from this account and suggest that the reader also considers the probability that a dominant or subordinate representation of a printed word will be instantiated. A potentially fruitful line of activity is to expand the model and make more formal and explicit assumptions that include variables such as the presence or absence of diacritics (see also [67]).

To summarize, the results reported replicate the word frequency, length and predictability effects in Arabic. The results also suggest that the subordinate bias effect can be observed when the disambiguation happens on the target word itself (not only when it is driven by information from prior context, as in previous research). The costs associated with the diacritics instantiating the subordinate representations of the targets affected all diacritized conditions, regardless of the target’s frequency or length (Exps. 1 and 2). Furthermore, we found no evidence that spotting the diacritics prior to fixating the target attenuated processing costs for low-predictability targets (Exp. 3). In fact, there was no evidence that spotting the diacritics prior to fixating the target facilitates the processing of the diacritized target, relative to when undiacritized, in any of the experiments. Further experimentation needs to be undertaken to replicate and expand upon the findings reported in this exploratory work. This will develop our knowledge regarding the relationship between diacritization and other word- and sentence-related variables, and accordingly serve to update current models and theories of word identification.

## Supporting information

S1 File Final GLMM models and pre-target analyses. The complete list of final models reported in the analyses, and analyses of pre-target region.
(DOCX)
