# Supplementary material for: Does diacritics‐based lexical disambiguation modulate word frequency, length, and predictability effects? An eye‐movements investigation of processing Arabic diacritics
Source: PLoS One. 2021 Nov 15;16(11):e0259987. doi: 10.1371/journal.pone.0259987 (PMC8592420; doi:10.1371/journal.pone.0259987)
Supplement: S1 File — The complete list of final models reported in the analyses, and analyses of pre-target region. (DOCX) [file pone.0259987.s001.docx]

S1 Supporting Information

1. Final reported models for target word and end of sentence regions

Exp. 1

Target word

Skipping

bi.lmer <- glmer(depvar ~ diac * word_length + (1 | pp) + (1 |stim), data = datafile, family = binomial)

First fixation duration

depvar.lmer = glmer(depvar ~ diac * freq + (1|pp) + (1|stim), control=glmerControl(optimizer="bobyqa",optCtrl=list(maxfun=12000)), data = datafile, family= "Gamma"(link = "identity"))

Single fixation duration

depvar.lmer = glmer(depvar ~ diac * freq + (1|pp) + (1|stim), data = datafile, family= "Gamma"(link = "identity"))

Gaze duration

depvar.lmer = glmer(depvar ~ diac * freq + (1|pp) + (1|stim), control=glmerControl(optimizer="bobyqa",optCtrl=list(maxfun=12000)), data = datafile, family= "Gamma"(link = "identity"))

Go past

depvar.lmer = glmer(depvar ~ diac * freq + (1 + diac * freq |pp) + (1 |stim), control=glmerControl(optimizer="bobyqa",optCtrl=list(maxfun=12000)), data = datafile, family= "Gamma"(link = "identity"))

Total fixation count

dv.lmer = lmer(depvar ~ diac * freq + (1 | pp) + (1 |stim), control=lmerControl(optimizer="bobyqa",optCtrl=list(maxfun=12000)), data = datafile)

Total fixation time

depvar.lmer = glmer(depvar ~ diac * freq + (1|stim), data = datafile, family= "Gamma"(link = "identity"))

End of sentence region

Go past

depvar.lmer = glmer(depvar ~ diac + (1+ diac |pp) + (1 |stim), data = datafile, family= "Gamma"(link = "identity"))

Exp. 2

Target word

Skipping

bi.lmer <- glmer(depvar ~ diac * word_length + (1 | pp) + (1 |stim), data = datafile, family = binomial)

First fixation duration

depvar.lmer = glmer(depvar ~ diac * word_length + (1|pp) + (1|stim), data = datafile, family= "Gamma"(link = "identity"))

Single fixation duration

depvar.lmer = glmer(depvar ~ diac * word_length + (1|pp) + (1|stim), data = datafile, family= "Gamma"(link = "identity"))

Gaze duration

depvar.lmer = glmer(depvar ~ diac * word_length + (1|pp) + (1|stim), data = datafile, family= "Gamma"(link = "identity"))

Go past

depvar.lmer = glmer(depvar ~ diac * word_length + (1|pp) + (1|stim), control=glmerControl(optimizer="bobyqa",optCtrl=list(maxfun=12000)), data = datafile, family= "Gamma"(link = "identity"))

Total fixation count

dv.lmer = lmer(depvar ~ diac * word_length + (1 | pp) + (1 |stim), control=lmerControl(optimizer="bobyqa",optCtrl=list(maxfun=12000)), data = datafile)

Total fixation time

depvar.lmer = glmer(depvar ~ diac * word_length + (1 + diac * word_length|pp) + (1 |stim), control=glmerControl(optimizer="bobyqa",optCtrl=list(maxfun=12000)), data = datafile, family= "Gamma"(link = "identity"))

End of sentence region

Go past

depvar.lmer = glmer(depvar ~ diac + (1+ diac |pp) + (1 |stim), data = datafile, family= "Gamma"(link = "identity"))

Exp. 3

Target word

Skipping

bi.lmer <- glmer(IA_SKIP ~ condition + (1 |stim), data = datafile, family = binomial)

First fixation duration

depvar.lmer = glmer(depvar ~ condition + (1+ condition|pp) + (1 |stim), control=glmerControl(optimizer="bobyqa",optCtrl=list(maxfun=12000)), data = datafile, family= "Gamma"(link = "identity"))

Single fixation duration

depvar.lmer = glmer(depvar ~ condition + (1+ condition|pp) + (1 |stim), data = datafile, family= "Gamma"(link = "identity"))

Gaze duration

depvar.lmer = glmer(depvar ~ condition + (1|pp) + (1|stim), control=glmerControl(optimizer="bobyqa",optCtrl=list(maxfun=12000)), data = datafile, family= "Gamma"(link = "identity"))

Go past

depvar.lmer = glmer(depvar ~ condition + (1|pp) + (1|stim), control=glmerControl(optimizer="bobyqa",optCtrl=list(maxfun=12000)), data = datafile, family= "Gamma"(link = "identity"))

Total fixation count

dv.lmer = lmer(depvar ~ condition + (1 | pp) + (1 |stim), control=lmerControl(optimizer="bobyqa",optCtrl=list(maxfun=12000)), data = datafile)

Total fixation time

depvar.lmer = glmer(depvar ~ condition + (1 + condition |pp) + (1 |stim), control=glmerControl(optimizer="bobyqa",optCtrl=list(maxfun=12000)), data = datafile, family= "Gamma"(link = "identity"))

End of sentence region

Go past

depvar.lmer = glmer(depvar ~ condition + (1|pp) + (1|stim), data = datafile, family= "Gamma"(link = "identity"))

2. Analyses of pre-target word

Exp. 1

There was no difference between first pass reading measures on the pre-target word between the target-diacritized and target-undiacritized conditions, in either of the two experiments. Specifically, in Experiment 1, single fixation duration on the pre-target word Mean target-diacritized = 257 ms (SD = 92), Mean target-undiacritized = 259 ms (SD = 106), , *t*s < 1, and gaze duration Mean target-diacritized = 322 ms (SD = 164), Mean target-undiacritized = 309 ms (SD = 150), b = -11.26, SE = 9.09, *t* < -1.24, *p* = .22.

Final reported models:

Single fixation duration

depvar.lmer = glmer(depvar ~ diac + (1+ diac|pp) + (1|stim), data = datafile, family= "Gamma"(link = "identity"))

Gaze duration

depvar.lmer = glmer(depvar ~ diac + (1+ diac|pp) + (1|stim), data = datafile, family= "Gamma"(link = "identity"))

Exp. 2

Similarly, in Exp. 2, single fixation duration on the pre-target word Mean target-diacritized = 250 ms (SD = 96), Mean target-undiacritized = 262 ms (SD = 96), *t* < 1; and gaze duration Mean target-diacritized = 302 ms (SD = 154), Mean target-undiacritized = 315 ms (SD = 166), *t* < 1. Of course, this absence of evidence that processing the diacritics on the target word affected the processing of the pre-target word (parafoveal-on-foveal, PoF, effects) does not rule out parafoveal processing of the diacritics (see e.g., Hermena et al., 2016).

Final reported models:

Single fixation duration

depvar.lmer = glmer(depvar ~ diac + (1+ diac|pp) + (1+ diac|stim), data = datafile, family= "Gamma"(link = "identity"))

Gaze duration

depvar.lmer = glmer(depvar ~ diac + (1+ diac|pp) + (1|stim), data = datafile, family= "Gamma"(link = "identity"))

Exp. 3

Similar to Exps. 1 and 2, we did not obtain any evidence that first pass reading measures on the pre-target word was affected by the presence of diacritics on the target. Specifically, single fixation duration on the pre-target word Mean target-diacritized = 293 ms (SD = 112), Mean target-undiacritized = 293 ms (SD = 122), and gaze duration Mean target-diacritized = 345 ms (SD = 158), Mean target-undiacritized = 340 ms (SD = 166), *t*s < 1.

Final reported models:

Single fixation duration

depvar.lmer = glmer(depvar ~ condition + (1+ condition|pp) + (1 |stim), control=glmerControl(optimizer="bobyqa",optCtrl=list(maxfun=12000)), data = datafile, family= "Gamma"(link = "identity"))

Gaze duration

depvar.lmer = glmer(depvar ~ condition + (1 + condition |pp) + (1 + condition |stim), control=glmerControl(optimizer="bobyqa",optCtrl=list(maxfun=12000)), data = datafile, family= "Gamma"(link = "identity"))
